# Growth factors in multiple myeloma: a comprehensive analysis of their expression in tumor cells and bone marrow environment using Affymetrix microarrays

**DOI:** 10.1186/1471-2407-10-198

**Published:** 2010-05-13

**Authors:** Karène Mahtouk, Jérôme Moreaux, Dirk Hose, Thierry Rème, Tobias Meißner, Michel Jourdan, Jean François Rossi, Steven T Pals, Hartmut Goldschmidt, Bernard Klein

**Affiliations:** 1 INSERM, U847, Montpellier, F-34197 France; 2 CHU Montpellier, Institute of Research in Biotherapy, F-34285 Montpellier, France; 3 Medizinische Klinik V, Universitätsklinikum Heidelberg, INF410, D-69120 Heidelberg, Germany; 4 Nationales Centrum für Tumorerkrankungen, INF350, D-69120 Heidelberg, Germany; 5 Université MONTPELLIER1, UFR Médecine, Montpellier, France; 6 Academic Medical Center, Department of Pathology, 1105 AZ Amsterdam, The Netherlands

## Abstract

**Background:**

Multiple myeloma (MM) is characterized by a strong dependence of the tumor cells on their microenvironment, which produces growth factors supporting survival and proliferation of myeloma cells (MMC). In the past few years, many myeloma growth factors (MGF) have been described in the literature. However, their relative importance and the nature of the cells producing MGF remain unidentified for many of them.

**Methods:**

We have analysed the expression of 51 MGF and 36 MGF receptors (MGFR) using Affymetrix microarrays throughout normal plasma cell differentiation, in MMC and in cells from the bone marrow (BM) microenvironment (CD14, CD3, polymorphonuclear neutrophils, stromal cells and osteoclasts).

**Results:**

4/51 MGF and 9/36 MGF-receptors genes were significantly overexpressed in plasmablasts (PPC) and BM plasma cell (BMPC) compared to B cells whereas 11 MGF and 11 MGFR genes were overexpressed in BMPC compared to PPC. 3 MGF genes (AREG, NRG3, Wnt5A) and none of the receptors were significantly overexpressed in MMC versus BMPC. Furthermore, 3/51 MGF genes were overexpressed in MMC compared to the the BM microenvironment whereas 22/51 MGF genes were overexpressed in one environment subpopulation compared to MMC.

**Conclusions:**

Two major messages arise from this analysis 1) The majority of MGF genes is expressed by the bone marrow environment. 2) Several MGF and their receptors are overexpressed throughout normal plasma cell differentiation. This study provides an extensive and comparative analysis of MGF expression in plasma cell differentiation and in MM and gives new insights in the understanding of intercellular communication signals in MM.

## Background

Multiple myeloma (MM) is a B cell neoplasia that affects 15 000 new patients per year in Europe and 15 000 in the United States. It is still an incurable disease with an average 5-year survival after high dose chemotherapy and autologous stem cells transplantation [1]. MM is characterized by the accumulation of a clone of malignant plasma cells in the bone marrow (BM). Hallmarks of MM are the presence of genetic abnormalities [2] and the dependence of tumor cells on their environment through cell communication signals [3]. Since the identification of IL-6 [4-6] and IGF-1 [7] as major myeloma growth factor (MGF) in 1988 and 1996, respectively, the identification of new autocrine and/or paracrine MGF has been constantly increasing, making it difficult to understand intercellular communication signals in MM (see [3,8] for review and Table 1A). This is however a major question, in particular with the aim to design novel targeted therapies for MM.

**Table 1:** List of growth factors investigated in the study.

**A. Growth factors that have been reported to be involved in myeloma biology**.		
**Growth factor**	**probe set**	References (*)

		
- Insulin-like growth factor-1 (IGF-1)	209541_at	[7,35-39]
		
- Hepatocyte growth factor (HGF)	210997_at	[41-46]
		
- Macrophage inflammatory protein1 α (MIP1α/CCL3)	205114_s_at	[53-56]
- Growth/differentiation factor 15 (GDF15)	221577_x_at	[24]
- pleiotrophin (PTN)	209466_x_at	[57,58]
- Brain derived neurotrophic factor (BDNF)	206382_s_at	[59]
		
**IL-6 family cytokines**		
- Interleukin 6 (IL-6)	205207_at	[4,5,19,33 -35,60-68]
		
- Ciliary neurotrophic factor (CNTF)	NA	[69]
- oncostatin M (OSM)	230170_at	" "
- Leukemia inhibitory factor (LIF)	205266_at	" "
- Interleukin 11 (IL-11)	NA	" "
- cardiotrophin-like cytokine factor1 (CLCF1)	219500_at	[70]
		
**Other interleukins**		
- interleukin 1β (IL-1β)	39402_at	[18]
- interleukin 10 (IL-10)	207433_at	[71,72]
- interleukin 15 (IL-15)	205992_s_at	[73]
- interleukin 21 (IL-21)	NA	[74]
		
**TNF family members**		
- tumor necrosis factor α (TNF-α)	207113_s_at	[75-78]
- B cell activating factor (BAFF/TNFSF13B)	223501_at	[9,79-82]
- A proliferation inducing ligand (APRIL/TNFSF13)	210314_x_at	" "
		
EGF family members (**)		
- amphiregulin (AREG)	205239_at	[17,28]
- heparin-binding EGF-like growth factor (HB-EGF)	203821_at	[17,83,84]
- neuregulin 1 (NRG1)	208241_at	[17]
- neuregulin 2 (NRG2)	206879_s_at	" "
- neuregulin 3 (NRG3)	229233_at	" "
- neuregulin 4 (NRG4)	242426_at	" "
		
**FGF family members**		
- fibroblast growth factor 2/basis FGF (FGF2/bFGF)	204422_s_at	[32,51]
		
**Wnt family members**		[47-49]
- Wnt5A	205990_s_at	
- Wnt10B	NA	
- Wnt16	224022_x_at	
		
**Jagged family**		[85-88]
- Jagged 1 (JAG1)	209099_x_at	
- Jagged 2 (JAG2)	32137_at	
		
**VEGF family**		
- Vascular endothelial growth factor A (VEGFA)	212171_x_at	[31,89-92]
		

**B. Members of the VEGF, FGF, and Wnt family**.		

**Growth factor**	**probe set**	
	
VEGF family (5 members)		
- VEGFA*	212171_x_at	
- VEGFB	203683_s_at	
- VEGFC	209946_at	
- VEGFD/FIGF	NA	
		
FGF family members (22 members)		
- FGF1	1552721_a_at	
- FGF2*	204422_s_at	
- FGF3	214571_at	
- FGF4	206783_at	
- FGF5	210311_at	
- FGF6	208417_at	
- FGF7	230918_at	
- FGF8	NA	
- FGF9	206404_at	
- FGF10	NA	
- FGF11	227271_at	
- FGF12	1562794_at	
- FGF13	205110_s_at	
- FGF14	231523_at	
- FGF16	NA	
- FGF17	NA	
- FGF18	231382_at	
- FGF19^#^	NA	
- FGF20	NA	
- FGF21	NA	
- FGF22	NA	
- FGF23	NA	
		
Wnt family members (19 members)		
- Wnt1	NA	
- Wnt2	205648_at	
- Wnt2B	NA	
- Wnt3	229103_at	
- Wnt3A	NA^Ψ^	
- Wnt4	208606_s_at	
- Wnt5A*	205990_s_at	
- Wnt5B	221029_s_at	
- Wnt6	71933_at	
- Wnt7A	210248_at	
- Wnt7B	NA	
- Wnt8A	224259_at	
- Wnt8B	NA	
- Wnt9A	NA	
- Wnt9B	NA	
- Wnt10A	223709_s_at	
- Wnt10B*	NA	
- Wnt11	206737_at	
- Wnt16*	224022_x_at	

**A**. (*) The list of references is not exhaustive, and includes the original or major studies. (**) Among all ten EGF-family members, only those having a heparin-binding domain are listed here. Indeed, only EGF-ligands that bind the HSPG syndecan-1 present on MMC can stimulate myeloma cell growth [17]. Probe sets noted "NA" were considered to be "non-informative" (see additional file 1).

**B**. * Indicates that the growth factor has already been reported to be involved in myeloma biology (they are also listed in table 1A). ^# ^FGF19 is the human orthologue of FGF15 and was cloned by homology to mouse FGF15. ^Ψ ^Indicates that no probe set was found for this gene. Probe sets noted "NA" were considered to be "non-informative" (see additional file 1).

In this study, we have used U133P 2.0 Affymetrix microarrays to analyse the expression of a large panel of MGF in BM aspirates from MM patients, in purified cell subpopulations present in the BM of those patients, *i.e *CD138+ multiple myeloma cells (MMC), CD14+ monocytes, CD15+ polymorphonuclear neutrophils (PMN) and CD3+ T cells, as well as in *in vitro*-generated bone marrow stromal cells (BMSC) and osteoclasts. We provide for the first time a comprehensive overview of growth factor expression in the different BM cell populations of patients with MM.

## Methods

### Patients and cell samples

Samples were obtained in agreement to the French and German ethical laws. MMC were purified from the BM of 131 patients with newly-diagnosed MM (median age, 59 years) after written informed consent was given. The study has been approved by the ethic boards of Heidelberg University and Montpellier University hospitals. According to Durie-Salmon classification, 14 patients were of stage IA, 24 of stage IIA, 76 of stage IIIA, 14 of stage IIIB, one had a plasma cell leukaemia and 2 were of undetermined stage. Human normal bone marrow samples were obtained from filtration residues of the bone marrow harvested from healthy donors for stem cell allograft after agreement of the Center of Biological Resources of the Montpellier University Hospital. Buffy coats of peripheral blood cells were purchased from the French Blood Center according to a written approved convention between French Blood Center and Montpellier University Hospital. Normal BM plasma cell (BMPC) and primary MMC were purified using using autoMACS with anti-CD138 MACS microbeads (Miltenyi-Biotec, Paris, France) as previously described [9]. For the isolation of peripheral blood memory B cells (MB), monocytes, NK and T cells were first removed using anti-CD14, anti-CD16 and anti-CD3 magnetic beads (Dynal), and MB cells were then positively selected using anti-CD27 MACS microbeads (Miltenyi Biotec). Polyclonal plasmablasts (PPC) were generated from purified peripheral blood CD19^+ ^B cells *in vitro*, as described [10,11]. Whole BM samples (WBM) were obtained from 75 of the 131 patients with MM whose MMC were purified. According to the Durie-Salmon classification, 12 patients were in stage IA, 15 in stage IIA, 45 in stage IIIA, three in stage IIIB. In addition, BM T lymphocytes (CD3+), monocytes (CD14+) and polymorphonuclear neutrophils (PMN, CD15+) were simultaneously purified from the BM of five of those 75 patients, as described previously [9]. Stromal cells (BMSC, n = 5) and osteoclasts (n = 7), which cannot be harvested in BM aspirates, were generated *in vitro *from the BM of MM patients, as described [9].

### Preparation of complementary RNA (cRNA), microarray hybridization and gene expression profiling analysis

Microarray experiments were performed at the Institute of Research in Biotherapy http://irb.chu-montpellier.fr/en/laboratories_microarray.html in the Montpellier University Hospital. RNA was extracted with the RNeasy Kit (Qiagen) or the SV-total RNA extraction kit (Promega) and Trizol (Invitrogen), in accordance with the manufacturer's instructions. Biotinylated complementary RNA (cRNA) was amplified with a double in-vitro transcription, according to the Affymetrix small sample labelling protocol vII (Affymetrix). The biotinylated cRNA was fragmented and hybridized to the HG-U133 Plus 2.0 GeneChip oligonucleotide arrays according to manufacturer's instructions (Affymetrix). Fluorescence intensities were quantified and analyzed using the GCOS software (Affymetrix). Arrays were scaled to an average intensity of 100. A threshold of 1 was assigned to values under 1. Gene expression data were analysed with our bioinformatics platforms: RAGE, http://rage.montp.inserm.fr[12] and "Amazonia!", http://amazonia.montp.inserm.fr[13].

All microarray data presented in this paper have been deposited in the ArrayExpress public database, under accession numbers E-MEXP-2360 for BMPC [11] and E-TABM-937 for B cells, PPC, MMC and environment population samples. We also used Affymetrix data of 22 normal BMPC samples and a cohort of 345 purified MMC from previously-untreated patients from the Arkansas Research Group (Little Rock) [14,15]. These data are publicly available in the NIH Gene Expression Omnibus (GEO) http://www.ncbi.nlm.nih.gov/geo/ under accession number GSE2658 for MMC samples and GSE5900 for 22 normal BMPC.

### Statistical analysis

For supervised analyses, differentially expressed genes were identified by a student t-test, and *p*-values were adjusted for multiple comparisons using the Benjamini and Hochberg correction. The threshold for significance was set to a *p *value ≤ 0.05. Among those genes, those with a fold change ≥ 2 were retained. Among the genes found to be statistically overexpressed with a fold change ≥ 2 in a given population, those with 100% absent call in this population were considered not to be biologically relevant and were removed. The call ("present" or "absent") is determined by Affymetrix GCOS-software and indicates whether a gene is reliably expressed or not. Regarding analysis on the different environment populations, whole bone marrow samples (WBM) were not included for supervised analysis. They include both MMC and environment cells and the expression of a given gene could be the reflection of its expression in MMC or in the environment or both.

## Results

### Affymetrix microarrays to investigate Myeloma Growth Factor (MGF) expression in BM subpopulations

Based on the literature data, we have listed 32 growth factors that have already been described as growth factors for MMC (Table 1A). As VEGFs, Wnts and FGFs are members of families with strong homologies, we extended the study to all members of those families, even if only some of them have already been shown to play a role in MM (Table 1B). Gene expression of 24/32 known MGF, 3/4 VEGFs, 13/22 FGFs and 11/19 Wnts could be interrogated with Affymetrix U133 Plus 2.0 arrays (Tables 1A and 1B). Other genes could not be studied due to a lack of probe set (one gene: Wnt3A) or to a "non-informative" probe set, *i.e*. with a low signal and an absent call in ≥ 95% of the samples (20 genes, see additional file 1). Thus 51 growth factors could be interrogated by 147 probe sets. When several probe sets were available for a given gene, the probe set with the highest percentage of "present call" among all samples or the one with the highest variance in case of 100% presence was selected. Probe sets are listed in Tables 1A and 1B.

### The majority of MGF are overexpressed in the tumor environment compared to MMC

**Figure 1:**
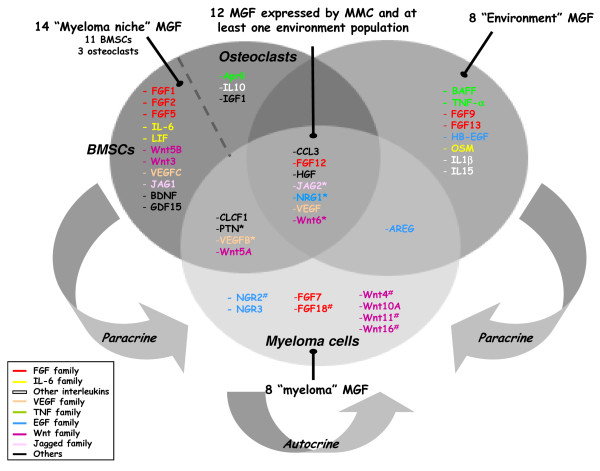
**The majority of MGF is expressed by the BM environment of patients with MM**. 42/51 MGF genes that are expressed in MMC and/or in the environment are represented in a Venn diagram according to their overexpression in each BM population compared to the others (p ≤ .05, fold-change ≥ 2). *Indicates that those 5 genes were expressed in MMC and environment cells, but without any change in signal between present or absent call (data not shown). This suggests that either those probe sets poorly work or their expression level is at the limit of sensitivity and thus can hardly be detected by Affymetrix probe sets. ^# ^Those genes were not statistically significantly overexpressed in MMC, but they displayed an absent call in all environment population (see additional file 2, table S-I) and they were thus defined as "myeloma MGF".

In order to identify which MGF are expressed by each cell component of the BM, we compared the expression of the 51 MGF genes among all BM subpopulations. 3/51 MGF genes - FGF7, NRG3, Wnt10A - were statistically significantly overexpressed in purified myeloma cells (MMC, n = 131) compared to each other BM population: T cells (CD3, n = 5), monocytes (CD14, n = 5), CD15+ polymorphonuclear neutrophils (PMN, n = 5), bone marrow stromal cells (BMSC, n = 5), and osteoclasts (n = 7) (p ≤ .05, fold-change ≥ 2). They were defined as "myeloma MGF" (Figure 1 and additional file 2, table S-I). 22/51 genes were overexpressed in cells from the environment compared MMC (p ≤ .05, fold-change ≥ 2). 14 out of those 22 MGF genes were overexpressed in cells of the putative tumor niche, *i.e *BMSC (11 genes) and osteoclasts (3 genes), compared to MMC and other BM subpopulations (CD3, CD14 and PMN). They were defined as "myeloma niche MGF" (Figure 1 and additional file 2, table S-II). 8/22 genes were overexpressed in at least one BM subpopulation (CD3, CD14 and/or PMN) compared to MMC, BMSC and osteoclasts (p ≤ .05, fold-change ≥ 2). They were defined as "environment MGF" (Figure 1 and additional file 2, table S-III). 17/51 genes were not classified by the supervised analysis. For the majority of them (12 genes), the reason is that they were highly expressed both in MMC and in at least one environment population (Figure 1 and additional file 2, table S-IV). The five remaining genes (NRG2, Wnt4, Wnt11, Wnt16 and FGF18) were considered as a "myeloma MGF" although they were not statistically significantly overexpressed in MMC compared to environment cell populations. Indeed, they displayed a present call in some of the MMC samples whereas they were absent in all other BM populations (see additional file 2, table S-I). Finally, 9/51 MGF were not/weakly expressed, neither in MMC nor in the environment. They had less that 5% of presence in MMC samples and BM subpopulations and they were not expressed in BMSC and osteoclasts (data not shown). Those genes include 5 members of the FGF family (FGF3,4,6,11,14), 3 members of the Wnt family (Wnt2A,7A,8A) and NRG4. This lack of expression could not be attributed to a lack of detection by a non-functional Affymetrix probe set, as expression of these genes could be detected using Affymetrix microarrays in various normal tissues (data not shown, see additional file 1). Altogether, those data indicate that the majority of MGF are overexpressed by the environment compared to MMC, and suggest that BMSC are the main source of MGF.

### MGF genes overexpressed in MMC compared to the environment

**Figure 2:**
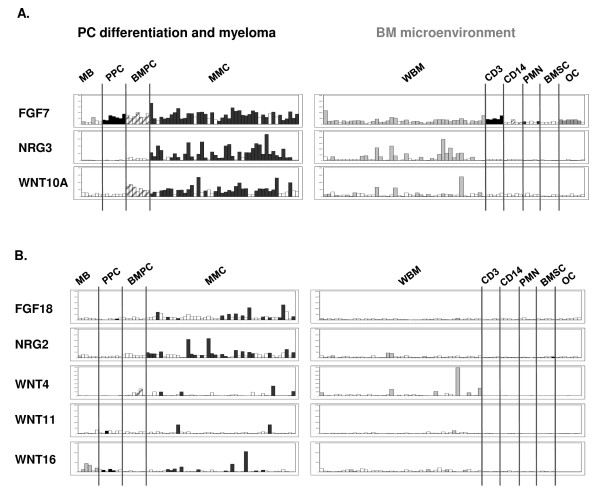
**Gene expression of "myeloma MGF" in bone marrow subpopulations**. (A) Overexpressed genes in MMC versus CD14, PMN, CD3, BMSCs and osteoclasts (p ≤ .05, fold-change ≥ 2). (B) MMC genes that were not statistically significantly overexpressed in MMC but displayed an absent call in all environment population (see additional file 2, table S-I). Histograms show the gene signal intensity on the Y axis as arbitrary units determined by the Affymetrix software. Each histogram features the same samples: 6 MB, 7 PPC, 7 BMPC, 44 MMC, 44 WBM, 5 CD3^+^/CD14^+^/PMN samples from newly diagnosed myeloma patients, 5 BMSC and 7 osteoclast samples generated *in vitro*. Expression data of 131 MMC samples and 75 WBM samples are presented in additional file 2. For clarity of the presentation, only 44/131 MMC and 44/75 WBM, both obtained from the same patients, are depicted here. White bars indicates that the gene displays an "absent" detection call. Abbreviations: MB, memory B cells; PPC, plasmablasts; BMPC, bone marrow plasma cells; MMC, myeloma cells, WBM, whole BM; CD3, T cells; CD14, monocytes; PMN, polymorphonuclear neutrophils; BMSC, stromal cells; OC, osteoclasts.

The 3 "myeloma MGF" as well as FGF18, NRG2, Wnt4, Wnt11 and Wnt16 (100% absent call in all environment populations) are represented in Figure 2. For clarity of the presentation, only 44/131 MMC samples as well as 44 whole BM samples (including MMC and cells of the environment; WBM), both obtained from the same patients, are depicted here. "Myeloma MGF" include 2 members of the EGF family (NRG2 and NRG3), 2 members of the FGF family (FGF7 and FGF18) and 4 members of the Wnt family (Wnt4, Wnt10A, Wnt11 and Wnt16). Except for FGF7, those 8 MGF displayed 100% absent call in all other BM cell populations (table S-I). Hence, the expression detected in the WBM samples is due to the MMC (Figure 2, right panel) and those factors are exclusively autocrine for the MMC. Three of these 8 genes - FGF7, Wnt4 and Wnt10A - were also expressed in normal plasma cells (Figure 2).

### MGF genes overexpressed in cells of a putative myeloma niche, *i.e *BMSC and osteoclasts, compared to MMC and other BM subpopulations

**Figure 3:**
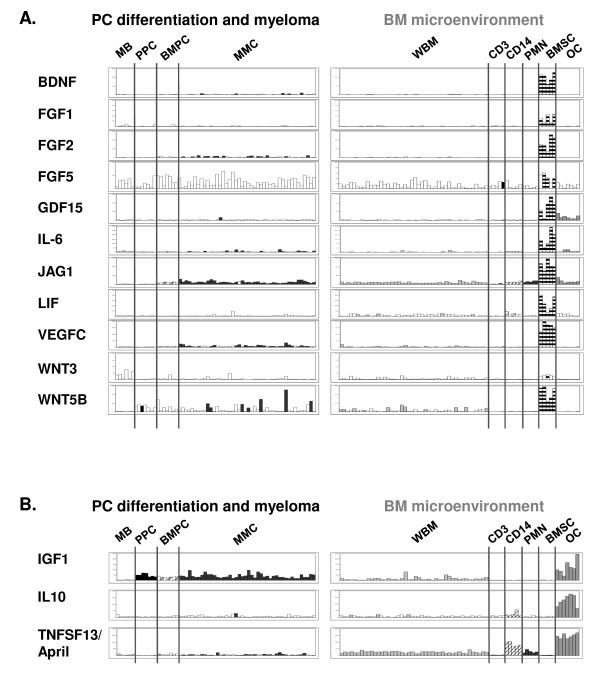
**Gene expression of "myeloma niche MGF" in bone marrow subpopulations**. Genes overexpressed in BMSCs (A) and in osteoclasts (B) versus CD14, PMN, CD3 and MMC (p ≤ .05, fold-change ≥ 2). See also legend of Figure 2.

The 14 "myeloma niche" MGF are represented in Figure 3. 11/14 genes were overexpressed in BMSC compared to all other BM populations (p ≤ .05, fold-change ≥ 2): BNDF, 3 members of the FGF family (FGF1, FGF2, FGF5), GDF15, IL-6, JAG1, LIF, VEGFC and 2 members of the Wnt family (Wnt3 and Wnt5B) (Figure 3A). 3/14 genes were overexpressed in osteoclasts compared to all other BM populations (p ≤ .05, fold-change ≥ 2): IGF1, IL10 and April (Figure 3B). IGF-1 and IL-6 have been extensively described as paracrine myeloma growth factors [5,6]. Accordingly, they belong to the "myeloma niche" category. Interestingly, IL-6 and IGF-1 were also expressed by the MMC themselves, in 60% and 100% of the patients, respectively (% of present call, table S-II), but at a lower level than in BMSC for IL-6 (median IL-6 expression in MMC = 55, versus 1867 in BMSC, table S-II) and in osteoclasts for IGF-1 (median IGF-1 expression in MMC = 424, versus 1104 in osteoclasts, table S-II).

### MGF genes overexpressed in cell subpopulations of the environment compared to MMC, BMSC and osteoclasts

**Figure 4:**
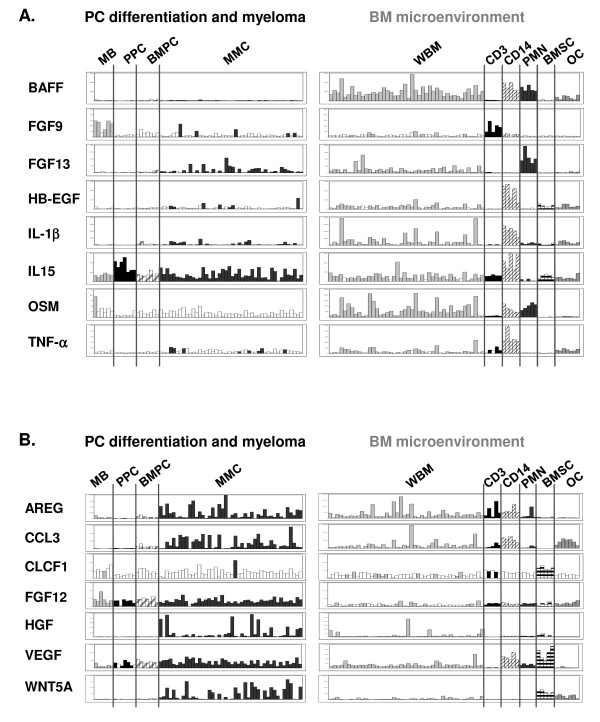
**Gene expression of "environment MGF", and MGF expressed both in MMC and in the environment**. (A) Genes overexpressed in at least one of the population CD14, PMN, CD3 versus MMC, BMSCs and Ocs (p ≤ .05, fold-change ≥ 2). (B) MGF genes that were not statistically significantly overexpressed in any of the BM populations. They were expressed in MMC and at least one the environment subpopulation. See also legend of Figure 2.

The 8 "environment" MGF are represented in figure 4A and listed in additional file 2, Table S-III. CD14-positive monocytes overexpressed 6/8 "environment" MGF (p ≤ .05, fold-change ≥ 2): 2 TNF-superfamilly members (TNFSF13B/BAFF and TNF-α), OSM, IL-1β, IL15 and HB-EGF. CD15-positive PMN also overexpressed BAFF and OSM, as well as FGF13, compared to MMC, BMSC and osteoclasts. Thus, monocytes and PMN may be an important source of growth factors for the tumor, especially because PMN represent around 50% of the cells in the BM [16]. CD3+ T cells overexpressed FGF9 (Table S-III).

### MGF genes expressed both by MMC and the environment

12/51 genes were not classified by the supervised analysis (Table S-IV). For seven of them, the reason is that they were highly expressed both in MMC and in at least one environment cell population: AREG, Wnt5A, CCL3, CLCF1, VEGF, FGF12 and HGF (Figure 4B and table S-IV). AREG was expressed in MMC and WBM samples, as well as in CD3, CD14 and PMN subpopulations, but it was weakly or not expressed by BMSC and osteoclasts. In contrast, Wnt5A was not expressed in WBM samples and in the subpopulations, but it was expressed in osteoclasts and BMSC. It should be noted that HGF expression in MMC (median = 218) was not significantly higher that that in BMSC (median = 194, p = 0.4), CD14 (median = 99, p = 0.1) and PMN (median = 102, p = 0.1). However, HGF expression in MMC among 131 patients was very heterogeneous and it could be very high in some patients' MMC (range 1 to 5378). Other 5 genes (PTN, NRG1, JAG2, VEGFB and Wnt6) were also expressed in MMC and environment cells, but without any change in signal between present or absent call (data not shown). This may suggest that those probe sets poorly work. It may also be that the expression level is at the limit of sensitivity and thus can hardly be detected by Affymetrix probe sets. This is likely the case for NRG1 whose gene expression measured by real-time PCR has been already documented in MM [17].

### Growth factor receptor expression in MMC

Because the aim of this study was to describe the expression of growth factors that sustain myeloma cell growth through paracrine or autocrine stimulation, we focused on the analysis of receptor expression in MMC only. However, we cannot exclude that MGF receptor expression by cells from the environment (data not shown) could indirectly influence myeloma cell growth as well. Among 48 MGF receptors that are listed in Table 2, 36 could be interrogated with Affymetrix microarrays. Half of the MGF receptor genes (18/36) were expressed in more than 50% of the MMC samples (present call, Table 2). Among them, 4 MGF receptor genes were expressed in 100% of the patients (IL-6ST, IL-10 RB, BCMA or Notch 2), 1 MGF receptor gene was expressed in 99% of them (IL-6R) and 5 MGF receptor genes were expressed in more than 90% of the patients (IL-10RA, TACI and Frz receptors 4,7 and 9). This indicates that the vast majority of the patients, if not all, have receptors for IL-6, IL-10, Baff/April and Wnt. 14/36 MGF receptor genes were expressed in 10-50% of the patients and 4/36 of them were expressed in less than 10% of the patients (table 2). CLCF1R was absent is 100% of the MMC samples and IL1R in 99% of them (in agreement with previous data [18]), suggesting that their respective ligands produced by the environment (see figure 4) have no biological relevance to MMC.

**Table 2:** Affymetrix expression of growth factor receptor genes in MMC.

MFG	MGF receptor	probeset	Median (range)	P (%)
			on MMC	on MMC
			(n = 131)	(n = 131)
				
- IGF-1	- IGF-1R	203627_at	19 (1-85)	32
- HGF	- c-Met	203510_at	78 (4-728)	50
- CCL3	-CCR1	205098_at	136 (26-2840)	78
- GDF15	-* not known*	---	---	---
- PTN	- RPTP-β/ζ/	204469_at	24 (1-1958)	48
- BDNF	- NTRK2	207152_at	84 (4-286)	77
				
IL-6 family				
**- IL-6**	**- IL-6R (gp80)**	**205945_at**	**502 (7-3657)**	**99**
	**- IL6ST (gp130)**	**212195_at**	**1681 (210-5680)**	**100**
				
- CNTF	- CNTFR	NA	---	---
- OSM	- OSMR	205729_at	25 (1-88)	58
- LIF	- LIFR	225575_at	16 (1-203)	15
- IL-11	- IL-11R	204773_at	61 (3-140)	26
- CLCF1	- CRLF1	206315_at	15 (2-91)	0
				
other interleukins				
- IL-1β	- IL-1R	202948_at	10 (1-63)	1
**- IL-10**	**- IL-10RA**	**204912_at**	**212 (24-912)**	**92**
	**- IL-10RB**	**209575_at**	**240 (98-536)**	**100**
- IL-15	- IL-15R	207375_s_at	142 (16-403)	55
- IL-21	- IL-21R	221658_s_at	6 (1-395)	5
				
TNF family				
- TNF-α	- TNFRSF1A	241944_x_at	63 (4-233)	66
				
- BAFF	**- TNFRSF13B/TACI**	**207641_at**	**266 (50-745)**	**95**
- APRIL	- TNFRSF13C/Baff-R	NA	---	---
	**- TNFRSF17/BCMA**	**206641_at**	**3794 (968-8301)**	**100**
				
EGF family	- ErbB1	211551_at	56 (6-148)	64
	- ErbB2	216836_s_at	30 (2-106)	5
	- ErbB3	202454_s_at	47 (79-172)	59
	- ErbB4	214053_at	51 (2-1091)	38
				
VEGF family	-VEGFR1/FLT1	NA	---	---
	-VEGFR2/FLK1	NA	---	---
	-VEGFR3/FLT4	NA	---	---
				
FGF family	- FGFR1	NA	---	---
	- FGFR2	NA	---	---
	- FGFR3	204379_s_at	4.2 (1-13160)	10
	- FGFR4	NA	---	---
				
Wnt family	- Frizzled1 (Frz1)	204451_at	75 (2-586)	34
	- Frz2	210220_at	30 (1-680)	30
	- Frz3	219683_at	80 (15-465)	19
	**- Frz4**	**218665_at**	**84 (2-348)**	**92**
	- Frz5	NA	---	8
	- Frz6	203987_at	229 (6-1425)	
	**- Frz7**	**203706_s_at**	**102 (12-2228)**	**98**
	**- Frz8**	**227405_s_at**	**34 (3-338)**	**93**
	- Frz9	NA	---	15
	- Frz10	NA	---	---
				
	- LRP5	NA	---	---
	- LRP6	225745_at	70 (1-137)	43
				
Jagged	- Notch1	218902_at	85 (24-233)	47
	**- Notch2**	**212377_s_at**	**216 (43-1743)**	**100**
	- Notch3	203238_s_at	28 (1.4-95)	2
	- Notch4	205247_at	40 (8-190)	35
				

MGF receptor genes expressed in more than 90% of the MMC samples are indicated in bold. MGF receptor genes expressed in less than 5% of the MMC samples are indicated in grey. Probe sets noted "NA" were considered to be not informative (see additional file 1).

### Expression of MGF and MGF receptors throughout normal PC differentiation and in MMC

In order to investigate the expression of MGF and MGF receptors throughout normal PC differentiation and in the generation of malignant plasma cells, we compared the expression of the 51 MGF and 36 MGF receptor genes in memory B cells (MB, n = 6), in *in vitro*-generated plasmablasts (PPC, n = 7) in purified bone marrow plasma cells (BMPC, n = 7), and MMC (n = 131). Data are shown in additional file 3, figures S-I and S-II and additional file 2, tables S-V and S-VI. The expression of 8 MGF and 10 MGF receptor genes was statistically significantly different between B cells and "PPC+BMPC" (p ≤ .05, fold-change ≥ 2). 4 MGF genes (OSM, Wnt6, Wnt16 and FGF9) were up-regulated in B cells, whereas others 4 (FGF7, CCL3, Wnt5B and IGF1) were up-regulated in "PPC+BMPC". 1 MGF receptor gene (IL10RA) was up-regulated in B cells, whereas other 9 genes were up-regulated in "PPC+BMPC" (p ≤ .05, fold-change ≥ 2): 3 FRZ-family members (FRZ1,3,6), the 2 members of the IL6R complex (IL-6R and IL-6ST), IL15RA, Met, TNFRSF17 and IL21R.

Interestingly, the expression of 12 MGF and 12 receptors was significantly different between PPC and BMPC, 11/12 MGF and 11/12 MGF receptor genes being up-regulated in BMPC compared to PPC (p ≤ .05, fold-change ≥ 2; figures S-I and S-II). Those 11 MGF genes include 2 Wnt-family members (Wnt5A, Wnt10A), 2 VEGF-family members (VEGFB and C), 1 FGF-family member (FGF2), 1 EGF-family members (AREG), 2 TNF-family members (Baff, April), PTN, CCL3 and JAG1. MGF receptor genes comprise 5 members of the FRZ family (FRZ1,2,6,7,8), IL6ST, NTRK2, Notch4, TNFRSF1A, CCR1 and LIFR. IL21R was upregulated in PPC. These data emphasize an increased expression of genes encoding for MGF and their receptors throughout plasma cell differentiation, in particular during late plasma cell stage, *i.e *in BMPC. It is noteworthy that FRZ receptor expression is a hallmark of PC differentiation.

We then compared the expression of the 51 MGF and 36 receptor genes in MMC and in BMPC. Only 3 MGF and 1 MGF receptor gene (FZD2) were significantly differentially expressed between MMC and BMPC (p ≤ .05, fold-change ≥ 2). FZD2 was overexpressed in BMPC, whereas all three MGF genes were overexpressed in MMC: AREG, NRG3 and Wnt5A. AREG and WNT5A were also upregulated in BMPC compared to PPC. In contrast, NRG3 was not expressed in any normal population (100% absent call) but it was expressed in 73% of the patients'samples with a very strong signal in some of them (see Figure 2). Those 3 MGF genes were also found to be overexpressed in MMC versus BMPC (p ≤ .05, fold-change ≥ 2) in an independent publicly available data set of 22 BMPC and 345 MMC patients' samples from the Arkansas Research Group (Little Rock, additional file 2, table SVII-A). Of note, 10/51 MGF and 5/36 receptor genes were "absent" in all BMPC samples (data not shown) whereas they were expressed in more than 10% (present call) of the MMC samples (see additional file 2). Hence, they may also be considered as being aberrantly expressed in MMC compared to normal BMPC although they are not statistically significantly overexpressed in MMC. Those MGF genes include HGF, 2 EGF-family members (HB-EGF and NRG2), 3 Wnt-family members (Wnt5B, Wnt11, Wnt16), 2 FGF-family members (FGF9, FGF18), BDNF and TNF-α (data not shown). MGF receptor genes include FGFR3, IL11R, IGF1R, FRZ4 and LRP6. Additional normal samples would be required to conclude about their relative expression in normal versus tumor cells. Among them, one MGF (HGF) and one MGF receptor (IL-11R) genes were statistically significantly overexpressed in MMC versus BMPC in the publicly available data set of 22 BMPC and 345 MMC patients' samples (p ≤ .05, fold-change ≥ 2; additional file 2, table S-VII).

## Discussion

Two major messages arise from this study. 1) The majority of MGF genes is expressed by the bone marrow environment. 2) Several MGF and their receptors are overexpressed throughout normal plasma cell differentiation.

22 out of the 51 MGF genes that could be interrogated by Affymetrix were significantly overexpressed by at least one BM environment population compared to others and to MMC, whereas only 3 of them were significantly overexpressed in MMC compared to the environment. These data emphasize the importance of the tumor microenvironment in MM pathogenesis. Purified primary MMC rapidly undergo apoptosis as soon as they are purified, suggesting that MGF produced by MMC themselves are not sufficient to maintain their survival [19]. The environment niche for MMC is not elucidated presently. It may resemble the normal plasma cell niche which comprises SDF-1-producing stromal cells and that is shared with hematopoietic stem cells and pre pro B cells [20]. Several studies have confirmed the importance of the endosteal cells, in particular BMSC (differentiated or not into osteoblasts) and osteoclasts to support myeloma cell proliferation and survival [21,22]. Other cells of the BM environment (CD14, CD3, PMN) and minor populations like plasmacytoid dendritic cells [23] could be important as well. In this study, we show that BMSC and osteoclasts are the main source of MGF. They highly express 11/21 and 3/21 "environment" MGF genes, respectively, in particular MGF that have been reproducibly identified in several studies: IGF-1, IL-6, APRIL, IL-10 [3]. In addition, these two cell types produce complementary sets of MGF, insuring that the endosteal niche could provide an optimal cocktail of growth factors for MMC survival. A point to emphasize is that MMC may progressively select a tumor-promoting environment. We have shown that BMSC from MM patients (MM-BMSC) support the growth of a stroma-dependent myeloma cell line better than normal BMSC and show a reduced matrix mineralisation capability [24]. Furthermore, BMSC from MM patients and from normal donors have a distinctive gene expression profile on microarrays analysis [24]. The fact that MM-BMSC have a specific gene signature although they have been cultured in vitro for several days in the absence of malignant plasma cells (cells are harvested after 41 days of culture for microarrays analysis [24]) suggest that cells harvested from the bone marrow microenvironment of myeloma patients keep their specific features even after in vitro culture. The second interesting finding is that MGF and MGF receptor gene expression is increasing progressively throughout normal plasma cell differentiation. 4 MGF and 9 MGF receptor genes were overexpressed in "PPC+BMPC" compared to B cells. 11 MGF and 11 MGF receptor genes were overexpressed in BMPC compared to PPC. This suggests that PC differentiation, and in particular the transition from immature plasmablast to mature plasma cell, is associated with a stronger dependence on growth factors. In agreement with this finding, it has been shown that normal plasma cells can survive and produce antibodies for very long periods as soon as they are located in the appropriate survival niches in the BM [25]. In contrast, 3 MGF genes only (AREG, NRG3 and Wnt5A) and none of the MGF receptor genes were significantly overexpressed in MMC compared to BMPC. One explanation is that MMC use the same growth factors than normal plasma cells. Accordingly, several growth factors for MMC also support the survival of normal plasma cells, like IL-6, BAFF and April [26,27], their specific receptors being expressed both in normal and malignant plasma cells (see table 2).

It was not in the scope of the study to validate the Affymetrix expression of all MGF. However, some of them have already been analysed by real-time RT-PCR and described by our group, like members of the EGF-family. Using real-time RT-PCR on MMC from 7 patients and U133A+B Affymetrix microarrays on a different cohort of 65 MM patients' samples, we have shown that AREG [28], NRG2 and NRG3 [17] are significantly overexpressed in MMC compared to their normal counterpart. This is in agreement with the data presented here (see Figure S-I). Previously, we also compared the expression of EGF-family members in MMC (n = 7 samples) and in the total BM microenvironment (n = 7 samples), using real-time RT-PCR. We have shown that NRG2 and NRG3 are mainly expressed by MMC whereas HB-EGF is mainly expressed by the environment [17]. Again, those results correlate with the current Affymetrix data (see figure 2 for NRG2 and NRG3; and figure 4A for HB-EGF). Other examples of formerly validated genes are Baff and April (Figures 4A and 3B), 2 members of the TNF superfamilly that were already shown to be highly expressed by the myeloma microenvironment compared to the MMC themselves by real-time RT-PCR [9]. Therefore, the good correlation between real-time RT-PCR analysis performed in independent series of samples and the Affymetrix expression profiles depicted here demonstrates the relevance of the present study and shows the power of microarrays to investigate as a whole the expression of MGF/MGFR genes in PC differentiation and MM.

Most interestingly, the microarrays analysis described in this paper uncovers new MGF and MGFR and/or describes for the fist time expression patterns of well known MGF. The new and most striking findings are discussed in the following.

**IL-6 **is an essential growth factor for myeloma cells [4,5]. Although it is now admitted that IL-6 is produced by the environment, it has been reported that IL-6 is produced by MMC [4] and that autocrine IL-6 production reflects a highly malignant phenotype [29]. Here we show that BMSC are the main producer of IL-6, in agreement with the literature data [30-32]. However, IL-6 can be produced by MMC in some patients (36% presence, see additional file 2, table S-II), but at a much lower extend compared to BMSC (fold-chance in expression between BMSC and MMC = 33.4). An important issue would be to understand the significance of this low autocrine IL-6 expression, compared to the huge amounts produced by BMSC. We have shown that a weak autocrine IL-6 production is sufficient to trigger cell cycling on myeloma cell lines (HMCL), whereas survival of those cells requires large exogenous IL-6 concentrations [33]. It is noteworthy that immature CD45^+ ^MMC express the IL-6 gene, unlike mature MMC [34]. In this population, autocrine IL-6 could be sufficient to trigger cell cycling, whereas the BM environment could be critical for triggering survival by producing other survival factors. In agreement with our previous findings [35], here we show that IL-6R and gp130 are expressed in 99% and 100% of the patients, respectively, indicating that the vast majority of the patients, if not all, is able to respond to IL-6 stimulation. We have recently shown that IL-6R expression in MMC is a bad prognostic factor due to its strong association with t(4:14) translocation [35]. Interestingly, both IL-6R and gp130 were overexpressed in normal plasma cells compared to B cells, in agreement with the known survival effect of IL-6 on plasma cells [26].

**IGF-1 **is an other essential growth factor for myeloma cells [7,35,36] and inhibition of the IGF-1-pathway reduces myeloma cell growth both *in vitro *and *in vivo *[37-39]. It has been speculated that BMSC would be an important source of IGF-1 in MM, probably based on the fact that murine stromal cells do secrete IGF-1 [40]. However, we show here that MM-BMSC do not/weakly express IGF-1. IGF-1 expression was 92-fold higher in osteoclasts compared to BMSC (see Figures 3B) and 4/5 BMSC displayed an absent call for IGF-1 (additional file 2, table S-II). In addition, MMC from all 131 patients expressed IGF-1. The presence of autocrine IGF-1 has already been reported in some HMCL [7], but this is to our knowledge the first study showing IGF-1 expression as a hallmark of primary MMC. IGF-1R had 100% absent call in BMPC whereas it was expressed in 32% of the MMC samples (see table 2), suggesting that functional IGF-1/IGF-1R autocrine loops are present in MMC of those patients. Recently, we have shown that IGF-1R expression in MMC delineates a group with adverse prognosis [35].

**HGF**. Several evidences suggest that the HGF/c-Met signalling pathway plays an important role in the biology of MM [41-44]. Here we describe for the first time the expression of c-Met in a large cohort of patients, and we have found that it was expressed in MMC from 50% of the patients. Although HGF expression in the environment (BMSC, CD14, PMN) was not statistically significantly different than that in MMC, it should be noted that HGF expression was very high in some patients' MMC (range 1-5318) suggesting that autocrine HGF loops are likely predominant in those high expresser patients. This is in agreement with elevated HGF levels found in the serum of some MM patients in association with advanced stages of MM and extended bone lesions [45,46]. It is noteworthy that HGF was overexpressed in MMC compared to BMPC in the public data set from the group of Shaughnessy (p ≤ .05, fold-change = 7.5, table S-VII). It did not reach the significance in our data set, probably because the number of samples is lesser.

**Wnt-family**. The expression of Wnt-family members and FRZ receptors has been documented in some myeloma cell lines [47] and in 4 primary MMC samples [48], and the biological relevance of this pathway in MM has been demonstrated [48,49]. In the present study, 8 Wnt were found in at least one BM subpopulation (MMC and/or environment): Wnt 3, 4,10A, 11, 16, 5A, 5B, 6. Noteworthy, Wnt5A was one of the 3 MGF significantly overexpressed in MMC compared to normal BMPC. All 7 FRZ receptors that could be interrogated with Affymetrix probe sets were expressed in some MMC samples and 3 of them (FRZ4, FRZ7, FRZ8) were expressed in more that 90% of the patients. LRP6, the required coreceptor of FRZ receptors, was expressed in 43% of the MMC samples. Altogether, those data indicate that Wnt/FRZ expression is a hallmark of MM. Dikkopf-1, an inhibitor of Wnt signaling expressed by primary MM cells, contributes to osteolytic bone disease by inhibiting the differentiation into osteoblasts [50]. Additional studies will be needed in order to understand the impact of DKK1 on the Wnt-induced proliferation of myeloma cells. Furthermore, we show that expression of several Wnt and FRZ is upregulated during normal plasma cell differentiation. Wnt5A and Wnt10 as well as FRZ1,2,6,7,8 were overexpressed in BMPC compared to PPC. FRZ1,3,6 were overexpressed in (PPC+BMPC) compared to B cells. Those data provide a rationale to investigate the role of the Wnt/FRZ family in the biology of normal plasma cell.

**FGF family**. Analysis of the FGF/FGFR family in MM has been mainly restricted to the angiogenic factor FGF2 (also called basic-FGF) and the nature of the cells producing FGF2 has remained controversial [32,51]. Here show that BMSC are the main source of FGF2. FGF2 was also expressed in 29% of the patients' MMC (38/131 present call, median expression = 25) in agreement with the data from Colla et al. [51], but the expression was 29.7-fold lower than that in BMSC. Other BM subpopulations (CD3, CD14, CD15) did not express FGF2 (see additional file 2, table S-II and figure 3A), as previously reported by Bisping et al. [32]. Furthermore, we show that other FGF-family members are broadly expressed in MM. Among the 13/22 FGFs that could be investigated with Affymetrix probe sets, 10 were expressed in at least one BM subpopulation (MMC and/or environment), suggesting that, like FGF2, they may directly or indirectly stimulate MMC survival and proliferation. Of note, it has been shown that only FGF2, 8 and 13 could be detected at the protein level on 12 HMCL whereas multiple FGFs were found at the mRNA level [52]. Although it was not in the scope of our study, FGF expression in MM should be validated at the protein level.

## Conclusion

In conclusion, this study provides an extensive description of MGF gene expression in the various cell populations of the BM of patients with MM, including MMC and the environment, and during normal plasma cell differentiation. We show an overexpression of MGF and MGFR genes during plasma cell differentiation, in particular in BMPC compared to PPC. Additionally, this study points out that the majority of MGF is expressed by the tumor environment compared to MMC. Some MGF are expressed by the MMC themselves, with a strong heterogeneity among patients. This study provides new insights in the understanding of the intercellular communication signals in MM, which is of major interest in order to design efficient biologically-based treatments for MM.

## List of abbreviations used

MM: multiple myeloma; MMC: myeloma cells; PPC: polyclonal plasmablasts; BMPC: bone marrow plasma cells; BM: bone marrow; BMSC: bone marrow stromal cells; PMN: polymorphonuclear neutrophils; MGF: myeloma growth factor; MGFR: myeloma growth factor receptor.

## Competing interests

The authors declare that they have no competing interests.

## Authors' contributions

KM designed research, performed the experiments, analyzed data and wrote the paper. DH, TM, JFR and HG collected bone marrow samples and clinical data. JM, DH, TR, MJ and STP analyzed data. BK is the senior investigator who designed research and wrote the paper. All authors read and approved the final manuscript.

## Pre-publication history

The pre-publication history for this paper can be accessed here:

http://www.biomedcentral.com/1471-2407/10/198/prepub

## Supplementary Material

Additional file 1 **Gene expression profiling analysis**. Material and methods describing how the so-called "non informative" probe sets were defined in the study. Click here for file

Additional file 2 **Expression of MGF genes in bone marrow cell populations and during normal plasma cell differentiation**. The file includes 7 tables showing the median expression and fold change in expression of MGF genes. Table S-I. Myeloma MGF genes. Table S-II. Myeloma niche MGF genes. Table S-III. Environment MGF genes. Table S-IV. Genes not statistically significantly overexpressed in cell populations. Table S-V. Expression of MGF during normal plasma cell differe. Table S-VI. Expression of MGF receptors during normal plasma cell differentiation and in MMC. Table S-VII. Expression of MGF and MGF receptors in BMPC and MMC samples from a public data set. Click here for file

Additional file 3 **Expression of MGF and MGF receptor genes during normal plasma cell differentiation and in MMC**. The file includes 2 figures that summarize the MGF (Figure S-I) and MGF receptor (Figure S-II) gene expression during normal plasma cell differentiation and in MMC. Click here for file
