# Supplementary material for: Growth factors in multiple myeloma: a comprehensive analysis of their expression in tumor cells and bone marrow environment using Affymetrix microarrays
Source: BMC Cancer. 2010 May 13;10:198. doi: 10.1186/1471-2407-10-198 (PMC2882921; doi:10.1186/1471-2407-10-198)
Supplement: Additional file 1 — Gene expression profiling analysis. Material and methods describing how the so-called "non informative" probe sets were defined in the study. [file 1471-2407-10-198-S1.PDF]

Additional file 1: Gene expression profiling analysis.

Material and Methods. 21 MGF and 12 MGF receptor genes (Tables 1 and 2) could not be studied owing to a lack of probe set (one gene: Wnt3A) or to a non-informative probe set. We considered a probe set to be non informative when it was characterized by an absent call in more than 95% of the samples studied here and more than 95% of a panel of 72 human normal tissues samples available from Hogenesch's group in a public database [1] and human embryonic stem cell samples [2] analysed with Affymetrix HG-U133A. The same global scaling and normalization was used for these and our data. The call (present or absent) is determined by Affymetrix GCOS software and indicates whether a gene is reliably expressed [3].

References.

1. Su AI, Wiltshire T, Batalov S, Lapp H, Ching KA, Block D, Zhang J, Soden R, Hayakawa M, Kreiman G, Cooke MP, Walker JR, Hogenesch JB: **A gene atlas of the mouse and human protein-encoding transcriptomes**. *Proc Natl Acad Sci USA* 2004, **101**(16):6062-6067.
2. Assou S, Lecarrour T, Tondeur S, Strom S, Gabelle A, Marty S, Nadal L, Pantesco V, Reme T, Hugnot JP, Gasca S, Hovatta O, Hamamah S, Klein B, De Vos J: **A meta-analysis of human embryonic stem cells transcriptome integrated into a web-based expression atlas**. *Stem Cells* 2007, **25**(4):961-73.
3. Liu WM, Mei R, Di X, Ryder TB, Hubbell E, Dee S, Webster TA, Harrington CA, Ho MH, Baid J, Smeekens SP: **Analysis of high density expression microarrays with signed-rank call algorithms**. *Bioinformatics* 2002, **18**(12):1593-1599.
