# Supplementary material for: Growth factors in multiple myeloma: a comprehensive analysis of their expression in tumor cells and bone marrow environment using Affymetrix microarrays
Source: BMC Cancer. 2010 May 13;10:198. doi: 10.1186/1471-2407-10-198 (PMC2882921; doi:10.1186/1471-2407-10-198)
Supplement: Additional file 2 — Expression of MGF genes in bone marrow cell populations and during normal plasma cell differentiation. The file includes 7 tables showing the median expression and fold change in expression of MGF genes. Table S-I. Myeloma MGF genes. Table S-II. Myeloma niche MGF genes. Table S-III. Environment MGF genes. Table S-IV. Genes not statistically significantly overexpressed in cell populations. Table S-V. Expression of MGF during normal plasma cell differe. Table S-VI. Expression of MGF receptors during normal plasma cell differentiation and in MMC. Table S-VII. Expression of MGF and MGF receptors in BMPC and MMC samples from a public data set. [file 1471-2407-10-198-S2.PDF]

Additional file 2: Expression of MGF genes in bone marrow cell populations and during normal plasma cell differentiation.

Table S-I. Myeloma MGF genes.

Table SI-A.

name	MMC n=131		WBM n=75		CD14 n=5		PMN n=5		CD3 n=5		BMSC n=5		Ocs n=7	
	**Median (range)**	**Nb P**	median	Nb P	median	Nb P	median	Nb P	median	Nb P	median	Nb P	median	Nb P
FGF7	**124 (10-480)**	**101**	57	64	29	1	42	1	84	5	29	0	75	6
NRG3	**190 (2-1088)**	**96**	47	39	13	0	6	0	13	0	4	0	4	0
WNT10A	**64 (1-269)**	**75**	21	24	32	0	28	0	30	0	11	0	17	0
FGF18	**35 (2-261)**	**27**	13	8	17	0	13	0	4	0	16	0	21	0
NRG2	**53 (3-338)**	**46**	19	7	9	0	10	0	12	0	17	0	14	0
WNT4	**6 (1-1057)**	**20**	12	16	1	0	1	0	1	0	1	0	1	0
WNT11	**12 (2-233)**	**10**	6	2	2	0	2	0	3	0	2	0	4	0
WNT16	**5 (1-212)**	**22**	5	7	4	0	2	0	3	0	4	0	1	0

Table SI-B.

name	MMC n=131	Subpopulations CD3, CD14, PMN	BMSC n=5	Ocs n=7
	**Median**	**FC**	**FC**	**FC**
FGF7	**124**	**2.5**	**4. 3**	**2.2**
NRG3	**190**	**14.6**	**47.5**	**47.5**
WNT10A	**64**	**2.1**	**5.8**	**3.8**

Genes overexpressed in MMC versus CD14, PMN, CD3, BMSCs and osteoclasts (Ocs) populations are listed. Genes in the upper part of the table of the table (FGF7, NRG3 and Wnt10A) were statistically significantly overexpressed in MMC versus other populations ($p \leq .05$, fold-change ≥ 2). Other 5 genes were not significant, but they displayed an absent call in all environment samples, and they were thus considered as “myeloma genes”. (A) Median expression and number of samples which display a “present call” (Nb P). Data from 131 MMC samples and 75 WBM samples are indicated here. Data from 44/131 MMC and 44/75 WBM samples, both from the same patients, are depicted in Figure 2. (B) Fold-change (FC) in expression in MMC versus other BM populations.

Table S-II. Myeloma niche MGF genes.

Table SII-A

name	BMSC n=5		Ocs n=7		MMC n=131		**WBM n=75**		CD14 n=5		**PMN n=5**		**CD3 n=5**	
	**median**	**Nb P**	**median**	**Nb P**	**median**	**Nb P**	**median**	**Nb P**	median	Nb P	median	Nb P	median	Nb P
11 BMSC GENES														
BDNF	**1089**	**5**	**3**	**0**	**13**	**9**	**3**	**1**	3	0	2	0	2	0
FGF1	**155**	**5**	**3**	**0**	**2**	**0**	**1.5**	**0**	2	0	2	0	7	0
FGF2	**742**	**5**	**4**	**0**	**25**	**38**	**7**	**3**	4	0	1	0	3	0
FGF5	**42**	**3**	**13**	**0**	**20**	**1**	**8**	**1**	7	0	7	0	12	1
GDF15	**755**	**5**	**137**	**7**	**22**	**2**	**14**	**1**	25	1	17	0	17	1
IL-6	**1867**	**5**	**107**	**7**	**55**	**47**	**28**	**28**	125	5	18	0	25	2
JAG1	**1636**	**5**	**216**	**7**	**214**	**127**	**144**	**74**	191	2	257	5	17	5
LIF	**238**	**5**	**37**	**0**	**8**	**1**	**16**	**6**	40	0	6	2	8	0
VEGFC	**1685**	**5**	**11**	**0**	**67**	**82**	**23**	**37**	17	2	27	0	23	0
Wnt3	**30**	**1**	**2**	**0**	**4**	**1**	**4**	**1**	1	0	2	0	2	0
Wnt5B	**540**	**5**	**2.6**	**0**	**77**	**43**	**39**	**21**	6	0	10	0	4	0
3 OSTEOCLAST GENES														
April	**31**	**5**	**2900**	**7**	**92**	**77**	**518**	**75**	1539	5	545	5	67	5
IGF1	**12**	**1**	**1104**	**7**	**424**	**131**	**113**	**70**	8	0	15	0	17	0
IL-10	**9**	**0**	**264**	**7**	**7**	**2**	**13**	**15**	39	4	10	5	19	5

Table SII-B

name	BMSC or OCs<br><br>Median	Subpopulations CD3, CD14, PMN<br>FC	MMC n=131<br>FC	Ocs or BMSC<br>FC
**11 BMSC GENES**				
BDNF	**1089**	**544.5**	**83.8**	**363**
FGF1	**155**	**51.7**	**77.5**	**51.7**
FGF2	**742**	**247.7**	**29.7**	**185.8**
FGF5	**42**	**5.3**	**2.1**	**3.2**
GDF15	**755**	**42**	**34.4**	**5.5**
IL6	**1867**	**68.1**	**33.4**	**17.2**
JAG1	**1636**	**8.7**	**7.6**	**7.6**
LIF	**238**	**15.9**	**29.8**	**6.3**
VEGFC	**1685**	**76.6**	**25.1**	**153.2**
WNT3	**30**	**15**	**7.5**	**15**
WNT5B	**540**	**90**	**7**	**180**
**3 OC GENES**				
April	**2900**	**5.3**	**31.5**	**93.5**
IGF1	**1104**	**78.9**	**2.6**	**92**
IL-10	**264**	**12.6**	**37.7**	**29.3**

Genes overexpressed in BMSC or osteoclasts Ocs versus CD14, PMN, CD3, MMC and Ocs/BMSCs, respectively, are listed (p ≤ .05, fold-change ≥ 2). (A) Median expression and number of samples which display a “present call” (Nb P). (B) Fold-change (FC) in expression in BMSC or Ocs versus other populations.

Table S-III. Environment MGF genes.

Table SIII-A.

name	**WBM n=75**		**CD14 n=5**		**PMN n=5**		**CD3 n=5**		MMC n=131		BMSC n=5		Ocs n=7	
	**median**	Nb P	**median**	Nb P	**median**	Nb P	**median**	Nb P	median	Nb P	median	Nb P	median	Nb P
BAFF	**1841**	75	**2751**	5	**2308**	5	**143**	5	72	106	78	5	772	7
FGF9	**31**	14	**48**	4	**17**	0	**175**	5	24	14	17	0	16	0
FGF13	**518**	74	**27**	1	**815**	5	**35**	2	92	91	15	0	44	5
HB-EGF	**144**	60	**1328**	5	**38**	0	**18**	0	60	13	272	4	139	3
IL-1β	**645**	75	**4289**	5	**512**	5	**67**	4	93	47	138	5	765	7
IL15	**107**	69	**444**	5	**21**	2	**122**	5	158	129	129	5	73	7
OSM	**484**	75	**662**	5	**1033**	5	**116**	5	30	4	4	0	80	4
TNF-α	**58**	54	**443**	5	**24**	0	**104**	3	46	13	7	0	115	7

Table SIII-B.

name	**CD14 n=5**	MMC	BMSC	OCS	**PMN n=5**	MMC	BMSC	OCS	**CD3 n=5**	MMC	BMSC	OCS
	**median**	FC	FC	FC	**median**	FC	FC	FC	**median**	FC	FC	FC
BAFF	**2751**	38.2	35.3	3.6	**2308**	32.1	29.6	3				
FGF9									**175**	7.3	10.3	10.9
FGF13					**815**	9	54.3	18.5				
HB-EGF	**1328**	22.1	5.9	9.5								
IL-1β	**4289**	45.6	31.1	5.6								
IL15	**444**	2.8	3.4	6.1								
OSM	**662**	22.8	165.5	8.3	**1033**	35.6	258.3	12.9				
TNF-α	**443**	9.6	55.4	3.9								

Genes overexpressed in at least in one of the population CD14, PMN, CD3 versus MMC, BMSCs and Ocs ($p \leq .05$, fold-change ≥ 2) are listed. (A) Median expression and number of samples which display a "present call" (Nb P). (B) Fold-change (FC) in expression in CD14, PMN or CD3 versus other BM populations.

Table S-IV. Genes not statistically significantly overexpressed in cell populations.

name	MMC n=131		WBM n=75		CD14 n=5		PMN n=5		CD3 n=5		BMSC n=5		Ocs n=7	
	median	Nb P	median	Nb P	median	Nb P	median	Nb P	median	Nb P	median	Nb P	median	Nb P
AREG	1201	131	610	75	1323	5	337	5	1150	5	60	5	64	5
CCL3	721	119	547	75	1880	5	162	5	608	5	6	0	2012	7
CLCF1	60	4	39	12	69	0	58	0	85	3	129	5	42	0
FGF12	59	114	19	35	33	5	36	5	37	4	27	5	38	6
HGF	218	106	89	71	99	5	102	5	7	0	194	5	36	7
JAG2*	69	39	29	29	75	5	55	1	84	5	49	3	48	5
NRG1*	42	19	6	2	39	1	19	0	27	1	27	1	26	0
PTN*	186	70	34	19	79	0	75	0	111	0	104	5	89	4
VEGF	374	131	296	75	1081	5	313	5	25	5	1654	5	35	7
VEGFB*	47	32	25	14	3	0	8	0	24	0	38	0	68	6
Wnt5A	339	103	60	53	13	2	10	1	10	0	585	5	311	7
Wnt6*	319	34	30	3	226	4	158	1	209	5	120	2	197	5

Genes that were not found to be statistically significantly overexpressed in one cell population compared to the others ($p > .05$ and/or ratio < 2) are listed. The table indicates the median expression and number of samples which display a "present call" (Nb P). * indicates genes (PTN, NRG1, JAG2, VEGFB and Wnt6*)* that were also expressed in MMC and environment cells, but without any change in signal between present or absent call (data not shown). This may suggest that those probe sets poorly work. It may also be that the expression level is at the limit of sensitivity and thus can hardly be detected by Affymetrix probe sets.

Table S-V. Expression of MGF during normal plasma cell differentiation and in MMC.

		Gene Name	**Fold-change**	median in MB	median in PPC	median in BMPC	median in MM
Comparison MB versus (PPC+BMPC)	Genes overexpressed in MB	OSM	**2.7**	65.5	17	41	30
		WNT6	**2.9**	555	153	230	319
		WNT16	**3.5**	33	20	4	5
		FGF9	**6.4**	251.5	32	70	23
	Genes overexpressed in PPC+BMPC	FGF7	**3**	42	114	134	124
		CCL3	**5.3**	53	138	736	721
		WNT5B	**10.3**	9.5	135	65	77
		IGF1	**12.2**	28.5	480	297	424
Comparison PPC versus BMPC	Genes overexpressed in PPC	IL15	**2.2**	129.5	303	139	158
	Genes overexpressed in BMPC	PTN	**2.0**	51	21	42	186
		VEGFB	**2.6**	63.5	25	65	47
		BAFF	**3.6**	104.5	30	107	72
		VEGFC	**3.7**	30	17	63	67
		WNT10A	**3.8**	38	30	115	64
		FGF2	**4.3**	13	6	26	25
		CCL3	**5.3**	53	138	736	721
		WNT5A	**6.3**	9.5	8	50	339
		AREG	**10.5**	218.5	31	326	1201
		JAG1	**12.0**	16.5	14	168	214
		APRIL	**12.8**	102	14	179	92
Comparison MMC versus BMPC	Genes overexpressed in BMPC	No gene					
	Genes genes overexpressed in MMC	AREG	**3.7**	218.5	31	326	1201
		NRG3	**3.9**	10	4	49	190
		WNT5A	**6.8**	9.5	8	50	339

A supervised analysis was performed in order to compare the MGF gene expression profile of memory B cells (MB) versus (PPC+BMPC), that of PPC versus BMPC and that of BMPC versus MMC. Genes differentially expressed genes between 2 populations in each comparison group are listed here ($p \leq .05$, fold-change ≥ 2). The median expression and fold-change are indicated for each gene, and genes are ranked according to the fold-change in each comparison group.

Table S-VI. Expression of MGF receptors during normal plasma cell differentiation and in MMC.

			foldchange	median in MB	median in PPC	median in BMPC	median in MM
Comparison MB versus (PPC+BMPC)	genes overexpressed in MB	IL10RA	2.8	929	294	428	212
	Genes overexpressed in PPC+BMPC	FZD3	2	24	45	52	80
		FZD1	2.7	25	48	92	75
		FZD6	3.2	30.5	60	138	229
		IL6ST	3.3	263.5	516	1220	1681
		IL15RA	3.5	58.5	179	251	142
		IL6R	6.8	64	816	564	502
		c-Met	7.1	5.5	60	19	78
		TNFRSF17	16.3	184.5	2525	3373	3794
		IL21R	27.3	4.5	244	58	6
Comparison PPC versus BMPC	Genes overexpressed in PPC	IL21R	4.2	4.5	244	58	6
	Genes overexpressed in BMPC	FZD1	2	25	48	96	75
		TNFRSF1A	2.0	48	42	85	63
		FZD6	2.3	30.5	60	138	229
		IL6ST	2.4	263.5	516	1220	1681
		NTRK2	2.4	91	45	107	84
		NOTCH4	2.4	59	29	69	40
		CCR1	3.4	76.5	74	248	136
		FZD7	3.7	30.5	35	128	102
		FZD8	3.9	67.5	12	47	34
		LIFR	12.0	5	3	36	16
		FZD2	70.7	9.5	3	212	30
Comparison MMC versus BMPC	Genes overexpressed in BMPC	FZD2	7	9.5	3	212	30
	Genes overexpressed in MMC	No gene					

A supervised analysis was performed in order to compare the MGF receptor gene expression profile of memory B cells (MB) versus (PPC+BMPC), that of PPC versus BMPC and that of BMPC versus MMC. Genes differentially expressed genes between 2 populations in each comparison group are listed here ($p \le .05$, fold-change ≥ 2). The median expression and fold-change are indicated for each gene, and genes are ranked according to the fold-change in each comparison group.

Table S-VII. Expression of MGF and MGF receptors in BMPC and MMC samples from a public data set.

Table SVII-A.

MGF genes	Median BMPC (n=22)	Median MMC (n=345)	**fold-change MMC/BMPC**
NRG3*	81.5	688.0	**8.4**
HGF	115.5	866.0	**7.5**
Wnt5A*	422.5	2183.0	**5.2**
AREG*	257.0	686.0	**2.7**
IL6	79.5	193.0	**2.4**

MGF receptor gene	Median BMPC (n=22)	Median MMC (n=351)	**fold-change MMC/BMPC**
IL11R	91.5	325.0	**3.6**

Table SVII-B.

MGF genes	Median BMPC (n=22)	Median MMC (n=345)	**fold-change BMPC/MMC**
WNT2	780.5	352.0	**2.2**
TNFSF13B	678.5	328.0	**2.1**

MGF receptor gene	Median BMPC (n=22)	Median MMC (n=351)	**fold-change BMPC/MMC**
IL1R	179	51	**3.5**

We used Affymetrix data of 22 normal BMPC samples and a cohort of 345 purified MMC from the Arkansas Research Group (Little Rock). These data are publicly available in the NIH Gene Expression Omnibus (GEO) under accession numbers GSE2658 and GSE5900. A supervised analysis was performed in order to compare the MGF and MGF receptor gene expression profile of BMPC and MMC samples ($p \leq .05$, fold-change ≥ 2). (A) Genes overexpressed in MMC versus BMPC. (B) Genes overexpressed in BMPC versus MMC. The median expression and fold-change are indicated for each gene.
