# Supplementary material for: Growth factors in multiple myeloma: a comprehensive analysis of their expression in tumor cells and bone marrow environment using Affymetrix microarrays
Source: BMC Cancer. 2010 May 13;10:198. doi: 10.1186/1471-2407-10-198 (PMC2882921; doi:10.1186/1471-2407-10-198)

Additional file 3: Expression of MGF and MGF receptor genes during normal plasma cell differentiation and in MMC.

Supplemental Figure S-I. Gene expression of MGF during normal plasma cell differentiation and in MMC. A supervised analysis was performed with MGF genes listed in Table 1 in order to compare the gene expression profile of memory B cells (MB) versus (PPC+BMPC), PPC versus BMPC and BMPC versus MMC. Only the differentially expressed genes between 2 populations in each comparison group are listed here (p ≤ .05. fold-change ≥ 2). The median expression and fold-change (in parenthesis) are indicated for each gene and genes are ranked according to the fold-change in each comparison group.

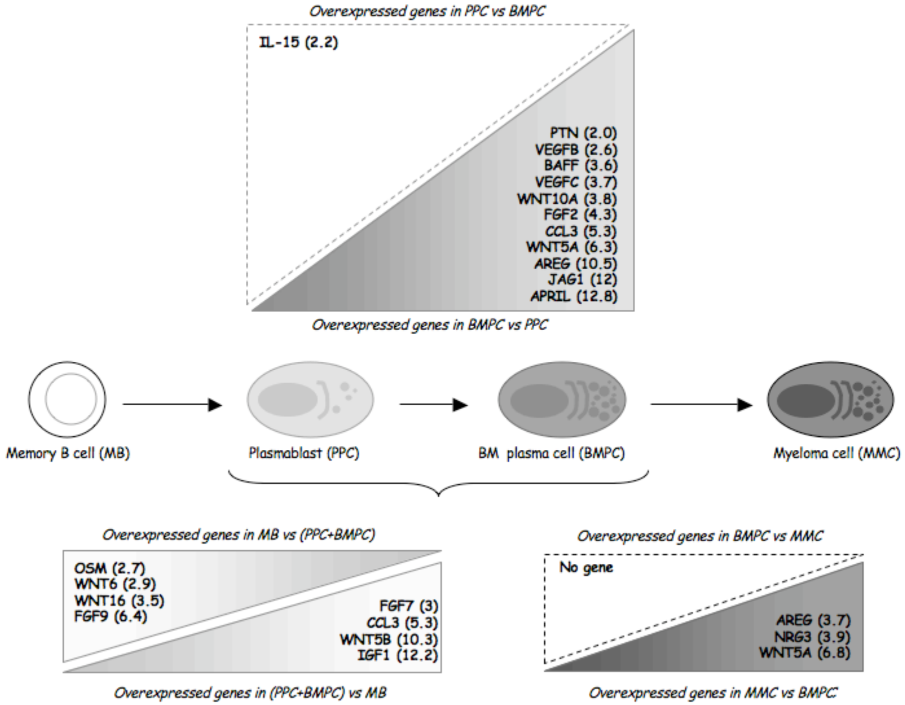

Supplemental Figure S-II. Gene expression of MGF receptors during normal plasma cell differentiation and in MMC. A supervised analysis was performed with MGF receptor genes listed in Table 2, in order to compare the gene expression profile of memory B cells (MBC) versus (PPC+BMPC), PPC versus BMPC and BMPC versus MMC. Only the differentially expressed genes between 2 populations in each comparison group are listed here ($p \leq .05$, fold-change ≥ 2). The median expression and fold-change (in parenthesis) are indicated for each gene, and genes are ranked according to the fold-change in each comparison group.

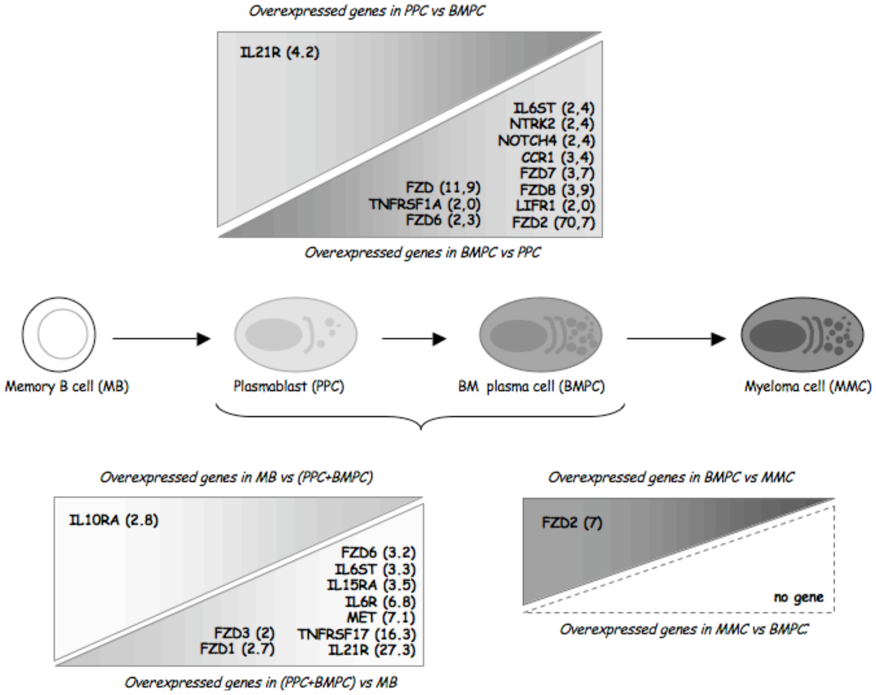

Supplement: Additional file 3 — Expression of MGF and MGF receptor genes during normal plasma cell differentiation and in MMC. The file includes 2 figures that summarize the MGF (Figure S-I) and MGF receptor (Figure S-II) gene expression during normal plasma cell differentiation and in MMC. [file 1471-2407-10-198-S3.PDF]
